# Multi-domain lifestyle intervention in older adults after myocardial infarction: rationale and design of the PIpELINe randomized clinical trial

**DOI:** 10.1007/s40520-023-02389-9

**Published:** 2023-03-25

**Authors:** Elisabetta Tonet, Andrea Raisi, Silvia Zagnoni, Giorgio Chiaranda, Rita Pavasini, Francesco Vitali, Federico Gibiino, Roberta Campana, Alberto Boccadoro, Antonella Scala, Luca Canovi, Veronica Amantea, Camilla Matese, Maria Letizia Berloni, Tommaso Piva, Valentina Zerbini, Laura Sofia Cardelli, Giovanni Pasanisi, Gianni Mazzoni, Gianni Casella, Giovanni Grazzi, Gianluca Campo

**Affiliations:** 1 Cardiovascular Institute, Azienda Ospedaliera Universitaria di Ferrara, Cona, FE Italy; 2 Center for Sports and Exercise Science, Department of Neuroscience and Rehabilitation, University of Ferrara, Ferrara, Italy; 3 Cardiology Unit, Ospedale Maggiore, Azienda USL Bologna, Bologna, Italy; 4 Sports Medicine and Health Promotion Unit, Azienda Unità Sanitaria locale di Piacenza, Piacenza, Italy; 5 Rehabilitation Cardiology, Azienda USL di Ferrara, Lagosanto, FE Italy; 6 Healthy Living for Pandemic Event Protection (HL-PIVOT) Network, Chicago, IL USA

**Keywords:** Older patients, Myocardial infarction, Exercise intervention, Lifestyle intervention

## Abstract

**Background:**

Traditional cardiac rehabilitation (CR) is effective in improving physical performance and prognosis after myocardial infarction (MI). Anyway, it is not consistently recommended to older adults, and its attendance rate is low. Previous studies suggested that alternative, early and tailored exercise interventions are feasible and effective in improving physical performance in older MI patients. Anyway, the demonstration that they are associated also with a significant reduction of hard endpoints is lacking.

**Aim:**

To describe rationale and design of the “Physical activity Intervention in Elderly patients with myocardial Infarction” (PIpELINe) trial.

**Methods:**

The PIpELINe trial is a prospective, randomized, multicentre study with a blinded adjudicated evaluation of the outcomes. Patients aged ≥ 65 years, admitted to hospital for MI and with a low physical performance one month after discharge, as defined as short physical performance battery (SPPB) value between 4 and 9, will be randomized to a multi-domain lifestyle intervention (including dietary counselling, strict management of cardiovascular and metabolic risk factors, and exercise training) or health education. The primary endpoint is the one-year occurrence of the composite of cardiovascular death or re-hospitalization for cardiovascular causes.

**Results:**

The recruitment started in March 2020. The estimated sample size is 456 patients. The conclusion of the enrolment is planned for mid-2023. The primary endpoint analysis will be available for the end of 2024.

**Conclusions:**

The PIpELINe trial will show if a multi-domain lifestyle intervention is able to reduce adverse events in older patients with reduced physical performance after hospitalization for MI.

**Trial registration:**

ClinicalTrials.gov NCT04183465.

## Introduction

Secondary prevention after myocardial infarction (MI) requires an integrated approach combining drugs (antithrombotic, anti-remodeling and lipid-lowering agents, beta-blockers), diet, smoking cessation, and measures to actively promote physical activity (home-based and/or supervised exercise intervention and/or cardiac rehabilitation program based on patient and acute event characteristics) [1]. The aging of the population, with the consequent increase in the mean age of patients admitted to hospital for MI, further stresses the importance of multi-domain cardiovascular (CV) secondary prevention. Older MI patients show several comorbidities and unrecognized but prognostically significant risk factors, such as low physical performance [2]. Older adults are the least fit and active group, and cardiovascular diseases accelerate physical deconditioning [3]. Low physical activity level is independently related to adverse events in patients with ischemic heart disease [4]. In addition, any hospital readmission further reduces the physical performance with potential prognostic worsening in terms of death [5]. Even though a multi-domain approach can be challenging in a specific subset of older patients, its value is undeniable, as shown by the FINGER (Finnish Geriatric Intervention Study to Prevent Cognitive Impairment and Disability) trial [6].

### Limitations of traditional cardiac rehabilitation programs in older MI patients

Many randomized clinical trials (RCT) and meta-analyses have clearly shown the benefit, in terms of hard endpoints, of the traditional cardiac rehabilitation (CR) program [7]. However, when putting traditional CR program in the context of older patients with MI, some points should be considered. The majority of these RCTs recruited young patients after cardiac surgery. Traditional CR consists of a high number of sessions and requires dedicated structures that are not so widely established. This also relates to low adherence and early withdrawal, which almost doubles the risk of recurrence of a CV event or death [8]. The rehabilitation sessions should start early after MI when patients are more receptive to lifestyle changes. Indeed, previous studies demonstrated that rehabilitation programs should start 3–4 weeks after the acute event, because in this phase MI patients are more open to accepting guidance about physical exercise to avoid future cardiac events [9, 10]. Retrospective studies suggested that for every day of delay between hospital discharge and cardiac rehabilitation, there is an associated 1% decrease in participation [9]. Pack et al. found a significant improvement in attendance in the group of patients with early appointments to cardiac rehabilitation centers [10]. Besides, standardized cardiac rehabilitation activities are generally not tailored to the needs of each patient, making them a limited recovery tool. Moreover, long-term maintenance of a healthy lifestyle and physical activity is not always the focus of these recovery programs, which seem to aim to more short-term recovery results. The lifestyle changes adopted during the rehabilitation period were probably not incorporated into a daily routine, hence hindering the much-needed life-long changes. This could be due to several factors such as the absence of a home-based program, difficult physical exercises, fear of falls and low motivation. Brovold et al. compared two groups of older patients undergoing physical activity, one group attending supervised physical exercise sessions, the other following a home-based prescription [11]. The results showed that supervised sessions determined a greater improvement in terms of lower limb function, but there were no significant differences among other outcomes such as physical functioning, mental health, social functioning and general health [11]. These data suggest that combining a home-based program and exercise sessions could be the best option.

### The proof-of-concept experience of the HULK trial

The “Physical Activity Intervention for Elderly Patients with Reduced Physical Performance after Acute Coronary Syndrome (HULK)” was a multicenter, investigator-driven, randomized clinical trial conducted at three Italian sites (NCT03021044). A detailed study outline has been previously published [12]. Briefly, 235 patients aged ≥ 70 years, hospitalized for acute coronary syndrome (ACS), and with Short Physical Performance Battery (SPPB) score between four and nine, were randomized to usual care and health education (control group) or to immediately start a physical activity (PA) intervention (intervention group) [12]. The PA intervention combined four supervised sessions (1, 2, 3, and 4 months after hospital discharge) with an individualized home-based PA program [12]. The general characteristics of the study population have been previously reported (mean age 76 [72–80] years, female sex 23%, more than 90% admitted for MI) [13, 14]. The attendance rate was high (72% [95%CI 64%–80%]) [13]. The exercise intervention was associated with significant improvements in daily physical activity starting from the first months after hospital discharge [13]. After 12 months, the patients of the interventional arm, as compared to those of the control one, showed higher SPPB values, a better quality of life (as measured by EuroQol-visual analogue scale), reduced perception of anxiety and/or depression, and lower occurrence of cardiac death and hospitalization for cardiac cause [14].

### Beyond the HULK study: rationale of the PIpELINe randomized clinical trial

The HULK study demonstrated that an early, tailored, and low-cost exercise intervention is safe and feasible in older patients hospitalized for ACS and effectively improves physical performance as assessed by SPPB score. However, the HULK trial has two major limitations. First, it mainly focused on exercise training, and it should not be considered a multi-domain intervention that may be further beneficial in the context of older MI patients. Second, it was underpowered for hard clinical endpoints. Although many previous studies have associated SPPB value with mortality and we found a reduction of adverse events at one-year follow-up, the HULK population was powered to appreciate significant changes in SPPB value, but findings regarding the outcome should be considered with caution. This is the starting point of the “Physical Activity Intervention in Elderly after Myocardial Infarction (PIpELINe)” randomized clinical trial. The aim of the PIpELINe trial is to investigate if an early, tailored and low-cost multi-domain lifestyle intervention combining exercise training, dietary counselling, and aggressive control of risk factors reduces CV death or rehospitalization for CV causes in older MI patients as compared to standard of care.

## Methods

### Study design

**Fig. 1:**
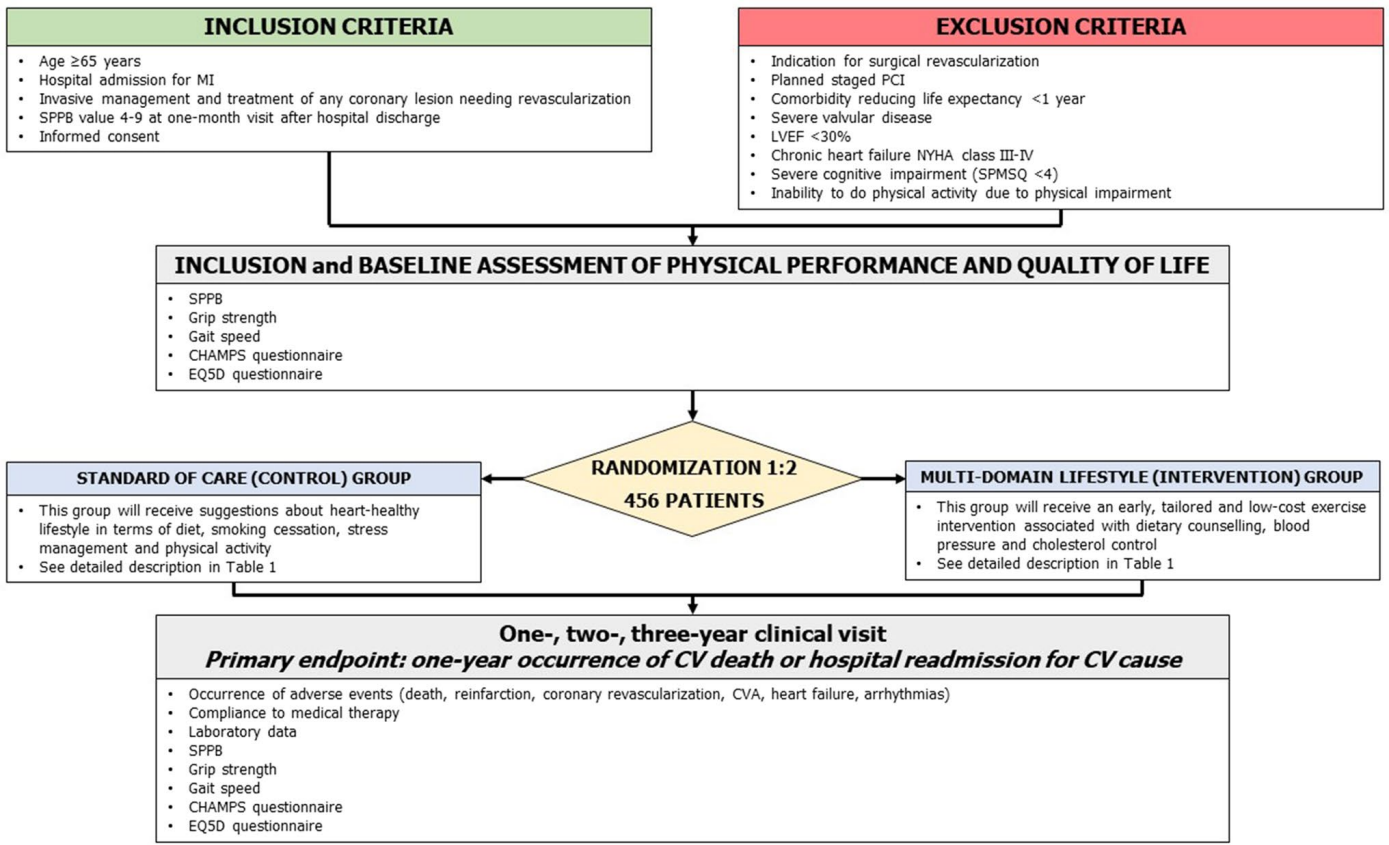
Inclusion and exclusion criteria and study design. MI: myocardial infarction. *SPPB* short physical performance battery, *PCI* percutaneous coronary intervention, *LVEF* left ventricular ejection fraction, *NYHA* New York Heart Association, *SPMSQ* short portable mental status questionnaire, *CHAMPS* Community Healthy Activities Model Program for Seniors, *EQ5D* Euro quality of life five domains, *CV* cardiovascular, *CVA* cerebrovascular accident

The PIpELINe trial is a prospective, randomized, multicenter, investigator-driven study with blinded adjudicated evaluation of outcomes (PROBE). The study flow chart, with inclusion and exclusion criteria, is reported in Fig. 1. The Steering Committee is responsible for the design and conduct of the study, study analyses, the drafting and editing of the paper and its final contents. The protocol has been approved by the institutional review boards in all participating centers.

### Screening, inclusion visit and randomization

**Fig. 2:**
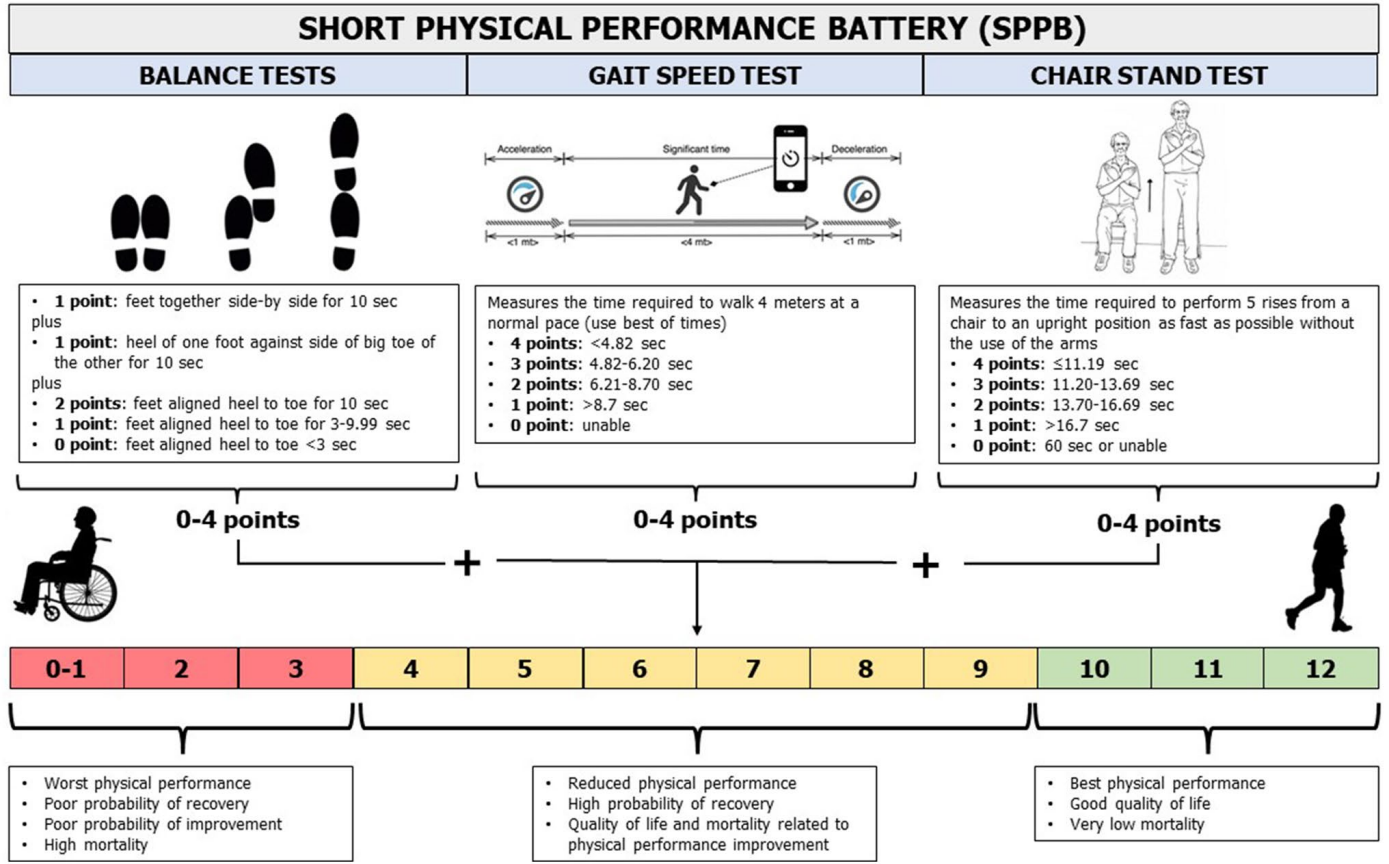
Description and interpretation of SPPB scale. *SPPB* short physical performance battery

All patients aged ≥ 65 years old admitted to hospital for MI and undergoing treatment of any coronary lesion considered suitable for revascularization will be provided information about the study protocol, aims, risks and benefits. MI diagnosis will be based on symptoms, electrocardiographic changes and rise and fall in cardiac biomarker (cardiac troponin), in agreement with current guidelines [15]. If the patient accepts to take part in the study and signs the informed consent, a study doctor will assess the SPPB. A brief description and interpretation of the SPPB test is reported in Fig. 2. Patients with an SPPB score of < 4 or > 9 will be excluded. Patients with an SPPB score of 4–9 are included in the run-in phase, and the study brochure will be provided. In the brochure, patients can find general recommendations about a heart-healthy lifestyle. One month after hospital discharge, during the inclusion visit (T1), the SPPB is re-evaluated and if the score is confirmed between four and nine, patients are randomized to multi-domain lifestyle intervention (intervention group) or the standard of care (control group) with a 2:1 allocation. Randomization is stratified by sex (male vs. female), SPPB value (4–6 vs. 7–9), clinical presentation (ST-segment elevation vs. no ST-segment elevation MI) and center. The key patient characteristics (i.e., inclusion/exclusion criteria, demographics, medical history, CV procedures, exams and treatments, laboratory test results) are recorded on the electronic Case Report Forms (eCRF, https://redcap.ospfe.it/).

### Standard of care (control) group

Patients randomized to the control group will be managed according to current guidelines [1]. Study procedures provided to this group during the inclusion visit are summarized in Fig. 1 and Table 1. Briefly, important advices for a heart-healthy lifestyle will be provided in terms of diet, smoking cessation, stress management and physical activity. Validated tools will be used to assess the quality of life, functional capacity, and physical activity.

**Table 1:** Study procedures in the control and intervention groups

Study group	Timing	Procedures
Control	Inclusion	Study Investigators perform a 20-min speech with patient and relatives about the major issues related to a heart-healthy lifestyle with some suggestions as follows: Healthy food choices: including vegetables, fruit, cereals, and water; avoiding foods containing saturated fat, foods and beverages with added sugars and salty foods; replacing saturated fat with monounsaturated and polyunsaturated fats from vegetables (oleic acid as in olive oil and rapeseed oil) and marine sources Weight control: to achieve and maintain a body mass index between 18.5 and 24.9 kg/m^2^ Smoking cessation Stress management: encouraging help from relatives and care givers Aerobic physical activity: aerobic physical activity such as walking and cycling; at least 20 min on 5 days/week should be engaged in physical activity; association of some calisthenic exercises, calibrating breathes and movements (description and teaching of 3 types of exercises) General recommendations: stairs rather than lift should be preferred; getting off the bus one stop earlier and walking for the rest of the way; going out for a walk with friends; for short trips, walking should be preferred Each participant receives dedicated brochures summarizing information and recommendations
Intervention	Inclusion (30 days after discharge)	Individual counselling with nutritionist Weight control Intensive management of CV and metabolic risk factors Dedicated smoking cessation programs Exercise intervention: the patient starts walking on the level at 2.0 km/h or lower according to perceived exertion for the first time. Every 30 s the study physician increases of 0.3 km/h up to a walking speed corresponding to a perceived exertion of 11–13 on the Borg scale for 1 km. Every 2 min, the study physician records the rate of perceived exertion Identification and description of the tailored home-based program Description and indication of exercises derived from Otago Exercise Program
	Subsequent study visits (60, 90, 180, 270 and 360 days after discharge)	Discussion about healthy food choice and compliance with diet Weight control Intensive management of CV and metabolic risk factors Discussion about compliance with medical therapy Exercise intervention: the patient starts walking at an updated intensity compared to the previous activity session. Every 30 s the study physician increases of 0.3 km/h up to a walking speed corresponding to a perceived exertion of 11–13 on the Borg scale for 1 km. Every 2 min, the study physician records the rate of perceived exertion Update and description of the tailored home-based program Description and indication of exercises derived from Otago Exercise Program

### Multi-domain lifestyle (intervention) group

All participants in the multi-domain lifestyle group will receive dietary counselling, strict management of CV and metabolic risk factors, and exercise training.

#### Dietary counselling

Dietary counselling will be provided by an experienced nutrition professional. Participants will attend, at inclusion, an individual face-to-face interview (60–90 min), where personal dietary goals and patient’s daily diet are agreed. The Mediterranean diet will be promoted as the healthiest type, and the following specific suggestions will be given: (i) to use olive oil as the main fat for cooking and dressing, (ii) to have at least 2 seasonal, fresh vegetable servings a day, (iii) moderate consumption of fresh fruit, (iv) consumption of fish at least 2 times per week, (v) favoring legumes to meat (strong limitation in the use of red meat) [16]. In addition, advice regarding food preparation is given, e.g., favoring cooking to frying and limiting the use of salt and sugar. A few examples of healthy meals (including information on ingredients and recipes) will be provided.

#### Management of CV and metabolic risk factors

The intensive management of CV and metabolic risk factors will be based on guidelines with aims to improve blood pressure, lipids, blood glucose, and to cease smoking [1]. At each study visit, study physicians will measure blood pressure and will supervise the patient’s blood pressure diary. The goal is to maintain blood pressure below 130/70 mmHg. In addition, laboratory data and body weight will be checked. Particular attention will be dedicated to cholesterol values, aiming to achieve in all patients the appropriate low-density lipoprotein (LDL) targets (< 55 or < 40 mg/dl) [1]. If necessary, study physicians will adjust and optimize the medical therapy. Smokers will be promptly supported with dedicated smoking cessation programs [17]. Compliance with medical therapy will be assessed at any study visits and monthly by phone call. The assessment of the compliance will be based on self-reports and further checked in pharmaceutical records as needed.

#### Exercise training

The exercise intervention consists of the early, tailored, mixed program investigated in the HULK study [12–14]. Briefly, the exercise intervention provides six supervised physical activity sessions (30, 60, 90, 180, 270 and 360 days after hospital discharge) and a series of exercises to be performed at home from the Otago Exercise Program, along with recommending at least 20 min of moderate walking. Figure 1 and Table 1 describe the procedures of the intervention group. An experienced team will supervise the exercise training. During the inclusion visit and during the following sessions, each patient will perform calisthenics exercises for ≈ 5 min, followed by the 1-km treadmill walking test (1k-TWT). A detailed description of the above-mentioned protocol is reported elsewhere [12]. Based on the results of the treadmill-walking practices, patients will be then encouraged to replicate similar walking sessions at home and outdoors, independently. The home program will be periodically adjusted during the subsequent visits.

### Follow-up visits

After hospital discharge, routine clinical follow-up is planned in all patients at 1, 6, 12 months, and yearly up to three years. At each visit, the clinical outcomes (death or readmission to hospital for any cause), compliance with medical therapy and smoking cessation, will be assessed. LDL, blood pressure and glycemic targets will be assessed. Validated questionnaires assessing the quality of life (EuroQol-5D, EQ-5D) and physical activity at home (Community Healthy Activities Model Program for Seniors, CHAMPS) will be administered. Physical performance will be assessed in terms of gait speed, handgrip and SPBB. Transthoracic echocardiography will be performed at discharge and during the one-year follow-up visit.

### Study endpoints

The primary endpoint is the one-year occurrence of CV death or hospital readmission for CV causes (Table 2). In case of repeated adverse events, the first that occurred will be the one considered. A detailed list of secondary endpoints is reported in Table 2. Being the study population based on older MI patients for whom also non-CV outcomes have a great relevance, careful attention will be deserved in the collection of all serious adverse events, including all-cause death and all-cause readmissions to hospital (Table 2). Follow-up will continue until 3 years after the randomization (Table 2). A committee consisting of clinicians who are blinded to treatment allocation (Clinical Event Committee) will review and adjudicate all adverse events based on the source documents.

**Table 2:** Study endpoints

	Timing	Endpoint
Primary	One-year	Cardiovascular death or hospital readmission for cardiovascular cause
Secondary	One-year	All-cause death
		Cardiovascular death
		Hospital readmission for cardiovascular cause
		Myocardial infarction
		Cerebrovascular accident
		Hospital readmission for any cause
		Quality of life (EQ-5D)
	Three-year	Cardiovascular death or hospital readmission for cardiovascular cause
		All-cause death
		Cardiovascular death
		Hospital readmission for cardiovascular cause
		Myocardial infarction
		Cerebrovascular accident
		Hospital readmission for any cause
		Quality of life (EQ-5D)

### Determination of sample size

Based on previous studies [13, 14, 18, 19], we may suppose a one-year primary outcome rate of about 25%. Previous studies showed a benefit from physical intervention ranging from 35 to 50%. Although the data should be considered with caution, we may suppose a reduction in the primary outcome of 40% (expected rate in the intervention arm 15%). Thus, based on alpha = 0.05 and beta = 80%, a total sample size of 435 patients is required. Estimating a possible loss of patients of 5% (*n* = 21), the final study population should be at least 456 patients, 152 in the control group and 304 in the intervention group.

### Statistical considerations

All statistical analyses will be performed by an independent statistician. The analyses will be performed on an intention to treat assumption, defined as all intentionally randomized patients, by randomization treatment. A detailed statistical analysis plan will be completed before the end of the study. In brief, continuous variables will be tested for normal distribution with the Kolmogorov–Smirnov test and with a visual estimate of *Q*–*Q* plot. Normally distributed variables will be presented as mean ± SD and compared by t-test and one-way ANOVA. Otherwise, median [inter-quartile range], Mann–Whitney *U* and Kruskal–Wallis tests will be used. Categorical variables will be summarized in terms of absolute and relative frequencies (percentages) and compared using the *χ*^2^ test. Statistical significance will be set at *α* = 0.05 level. Formal type-I error control will be ensured for the primary and the key secondary endpoint by a sequential procedure where significance for the key secondary endpoint is accepted only if the primary endpoint is positive. Kaplan–Meier curves will be plotted to describe freedom from adverse events, and difference between groups will be tested with the log-rank test. When appropriate, 95% CI will be calculated.

### Predefined substudies

The PIpELINe trial program includes some predefined sub-studies. The first one focuses on depression and its relationship with physical activity. The others are focused on specific biomarkers related to mitochondrial function, immunosenescence, inflammation, and endothelial function.

### State of the art

The PIpELINe trial is an investigator-initiated study, and the sponsor is the University Hospital of Ferrara. The sponsor received a research grant from the Italian Health Minister (Ricerca Finalizzata 2018, GR 2018-12367114). The study was approved on May 22th, 2019 by the Ethic Committee of the coordinating center (Comitato Etico Area Vasta Emilia Centro, Bologna, Italy). The study was registered on December 3rd, 2019, with the ClinicalTrials.gov Identifier NCT04183465. The enrollment phase started on March 27th, 2020. The date of the start of the recruitment was warmly recommended by the Italian Health Minister that partially supported the conduction of the study. It was concomitant to the first wave of the COVID-19 pandemic. The pandemic dramatically affected the recruitment in the 2020 and in the first 6 months of the 2021, due to reduction in the hospital admission for MI [20] and patient reticence to be involved in clinical studies during the pandemic. Then, we can assume that the pandemic has extended patient recruitment by about 12 months as compared to the original plan. We estimated to complete the enrollment in September 2023. Data for the primary endpoint evaluation will be available in September 2024. The follow-up will continue for up to three years.

## Discussion

Older MI patients have been less considered in previous and recent studies of secondary prevention [21]. In a preliminary study, Marchionni and colleagues compared traditional vs. home-based CR in patients ranging from 46 to 86 years [22]. The Authors found that both models were effective, and improvements were similar in middle-aged and old persons [22]. Anyway, the study was based on the assessment of work capacity and health-related quality of life, without any information regarding hard clinical endpoints [22]. Prabhakaran et al. randomized 3959 MI patients to a yoga-based rehabilitation program or educational advice [23]. They demonstrated that the yoga-based program improved patients’ health and return to their pre-infarct activities, but the study enrolled only a small percentage of older adults (8% in Yoga-CaRe group vs. 8.6% in Enhanced Standard care) [23]. Bush et al. investigated the effects of cardiac rehabilitation on hospitalization in older MI Medicare beneficiaries: cardiac rehabilitation initiators showed a lower rate of recurrent MI, CV and all-cause hospitalization when compared with non-initiators [24]. However, these interesting data were limited by the non-randomized nature of the study [24]. Another point to be noted concerns the number of patients in the two groups: of 32,851 subjects, only 6916 (21%) attended cardiac rehabilitation, confirming the low participation of older adults in rehabilitation activities [24]. The PIpELINe trial, starting from the above-mentioned limitations, will try to draw definitive conclusion regarding the best multi-domain lifestyle intervention for the secondary prevention of older MI patients. Data from the HULK study reported a satisfying rate of compliance and the effectiveness of the tailored exercise intervention in terms of functional capacity and quality of life [13, 14, 25]. The PIpELINe trial will expand the findings of the HULK study integrating the exercise intervention with a more complete multi-domain intervention. In addition, the PIpELINe trial is powered for hard endpoints and will allow us to clarify whether in older MI patients, a multi-domain lifestyle intervention may reduce the cumulative occurrence of CV death or hospital readmission for CV causes.

## Data Availability

The data that support the findings of this study will be available from the corresponding author, [GC], upon reasonable request.
